# Variations in Label Information and Nicotine Levels in Electronic Cigarette Refill Liquids in South Korea: Regulation Challenges

**DOI:** 10.3390/ijerph120504859

**Published:** 2015-05-05

**Authors:** Sungroul Kim, Maciej L. Goniewicz, Sol Yu, Bokyeong Kim, Ribhav Gupta

**Affiliations:** 1 Department of Environmental Health Sciences, Soonchunhyang University, Asan-city 336-745, South Korea; E-Mails: solsol0914@gmail.com (S.Y.); qhrud0309@gmail.com (B.K.); 2 Division of Cancer Prevention and Population Sciences, Department of Health Behaviors, Roswell Park Cancer Institute, Buffalo, NY 14263, USA; E-Mails: maciej.goniewicz@roswellpark.org (M.L.G.); ribhav.gupta97@gmail.com (R.G.)

**Keywords:** electronic cigarettes, labels, nicotine, regulation

## Abstract

Background: In South Korea, the consumption of liquid nicotine used in electronic cigarettes has dramatically increased from 4310 L in 2012 to 7220 L in 2013. This study aimed to examine the level of heterogeneity of contents of the labels and discrepancy of the nicotine content between that indicated on the label and the actual values for electronic cigarette liquid refill products in South Korea. Methods: We purchased 32 electronic cigarette liquid refill products (17 Korean domestic, 15 imported ones) and one pure nicotine product at six different electronic cigarette retail stores in Seoul between May and June 2014. The actual nicotine concentrations of each product were measured by a blinded analyst at Roswell Park Cancer Institute, Buffalo, NY, USA. Results: Three out of 15 imported liquid refill products provided manufacturing dates, while expiration dates were available on eight products. The range of nicotine concentration was from “not detected” to 17.5 mg/mL. Labeling discrepancies of the concentrations ranged from −32.2% to 3.3% among electronic cigarette liquid refill products. The highest concentration (150.3 ± 7.9 mg/mL) was found in a sample labeled as “pure nicotine”. Conclusions: There is no standardization of labelling among electronic cigarette liquids sampled from retail stores and the labels did not accurately reflect the content. One product labeled “pure nicotine” raises concerns, since it may be poisonous to consumers, especially to children. This study revealed the urgent need for the development of product regulations in South Korea.

## 1. Introduction 

In South Korea, according to statistics announced in 2014 by the Ministry of Health and SK Securities Co., Ltd. (Seoul, South Korea), one of major companies analyzing domestic and overseas stock markets and conducting business analysis independently from government and the tobacco and electronic cigarette (e-cig) industries, the consumption of the liquid nicotine used in e-cigs had dramatically increased, rising from 4310 L in 2012 to 7220 L in 2013 [1].

With consideration of the increase in the consumption of liquid nicotine-using e-cigs in south Korea as well as other countries, standards on quality control for nicotine concentration should be immediately implemented [2]. In South Korea, these products are currently imported from Europe, America, and China, and sold in retail shops or through the internet. Korean domestic brands of fluid products are also sold together with imported products. However, no regulations concerning labeling standards for the fluid products used in e-cigs in South Korea have been implemented. In contrast, the U.S. Food and Drug Administration (FDA) regulates e-cigs sold for therapeutic purposes although FDA cannot regulate e-cigs as medical devices [3]. Some U.S.-based e-liquid manufacturing companies have formed an industry group that has been developing labeling standards, suggesting that nicotine concentrations be ±10% of the value indicated on the label [4]. However, in South Korea, no such regulations exist yet.

Previous studies have evaluated the nicotine contents of e-cig liquid refill products and reported the differences between the actual measured nicotine contents and the amounts listed on the labels [5,6]. Those studies were conducted mainly in Europe or the U.S. Studies conducted in Asia, where the ways of vaporing liquid refill products may be different from those on other continents, are still limited [7].

In this study, we examined the heterogeneity of labelling of imported and domestic e-cig liquid refill products and the discrepancies between the nicotine contents indicated on the labels and the actual measured values for purchased e-cig liquid refill products.

## 2. Methods

### 2.1. Sample Collection

We purchased 32 e-cig liquid refill products (17 Korean domestic, 15 imported ones) and one pure nicotine product at six different e-cig retail stores with convenience based samplings in Seoul and Asan, South Korea between May and June 2014. Seventeen products were considered domestic, as they were labeled “Made in Korea”. The rest (n = 15) were imported from the United States (USA), Italy, the Netherlands, or China. Since there are some 200 products with different flavors, for convenience, we selectively purchased liquid products in cigar or tobacco flavors and the flavors that were most popular at each store, according to the store clerks. These data were informally collected from the store clerks by our research assistants in conversation with them. We summarized the names of the 32 refill liquids as well as countries of manufacture, nicotine content, manufacture dates, expiration dates, and any health warning statements on them. Labeling discrepancies between the nicotine content indicated on the label and the actual measured values were calculated as percentage differences.

### 2.2. Measurement of Nicotine Concentration

To measure the nicotine in each product, aliquots of approximately 4 mL were taken from each sample, stored in 15 mL amber vials, and shipped to the Roswell Park Cancer Institute, Buffalo, NY, USA, in four blank amber vials. Details of the analytical procedure can be found elsewhere [2,8]. Briefly, samples of each product (100 μL) were collected from each original bottle using the reverse pipetting technique. The samples were diluted with methanol (10 mL), and an internal standard (100 μL of 50 mg/mL quinolone solution in methanol) was added. The samples were then vigorously shaken for 10 min and subsequently analyzed as described below. The nicotine concentration was determined in triplicate (three measurements of the same sample) by a blinded analyst using gas chromatography with a thermionic specific detector (GC-TSD, Varian Inc., Walnut Creek, CA, USA) at the Roswell Park Cancer Institute. Calibration/control solutions were prepared and analyzed repeatedly during the instrumental analysis process [8]. For the nicotine separation, a HP-5, 30 m × 0.32 mm × 0.25 mm capillary column (Santa Clara, CA, USA) with flow rate of helium of 2.4 mL/min was used. A calibration curve was generated to cover the range of nicotine concentration from 0 to 250 mg/mL. To ensure accurate results for the samples each calibration curves had linear coefficients of 0.99 (R^2^ ≥ 0.99) or above. The average nicotine recovery was 102% and the precision of the method was 18%, and the lower quantitation limit and the limit of detection were 0.05 and 0.01 mg/mL, respectively [2]. 

## 3. Results

Information on the labelling of the 32 sampled liquids (Table 1) showed wide variation in all labelling elements; a lack of information was common in the domestic and the imported liquids. Health warnings varied widely in content and were less common in Korean than imported liquids.

The nicotine concentrations as labeled on the bottles and their corresponding measured concentrations are summarized in Table 2. Nicotine concentrations in the analyzed samples varied significantly from “none detect” to 17.5 mg/mL. Labeling discrepancies, calculated as percentage differences, ranged from −32.2% to 3.3% and measured nicotine concentrations were statistically significantly lower than the labeled levels (*p* < 0.01). The highest concentration (150.3 ± 7.9 mg/mL) was in a sample labeled “pure nicotine.” None of the domestic brands reported nicotine content and we could not detect nicotine in the domestic brands.

**Table 1:** Description of the liquid e-cig products, including countries of origin, manufacturers, suppliers, flavors, manufacturing and expiration dates, and types of health warning statement.

No.	Country of Origin	Manufacturer	Supplier	Flavor	Manufacturing Date	Expiration Date	Type of Health Warning Statement
1	China	None	None	Iris Melon	None	2015. 05. 27	None
2	China	None	None	The Black	None	2016. 05. 19	None
3	China	None	None	Korean Mini	None	None	1, 2
4	China	None	None	USA Mix	None	None	1, 2
5	Italy	Ritchy Group Ltd.	None	Citrus Mix	None	2015. 12	3, 4, 5
6	Italy	Ritchy Group Ltd.	None	American Blend	None	2016. 03	3, 4, 5
7	Italy	Ritchy Group Ltd.	None	Cuban Cigar	None	2016. 03	3, 4, 5
8	Netherlands	Cignit Korea	Janty Netherland	Cigar	None	2016. 04. 18	None
9	USA	America’s Smoke Juice	Korea Electronic Cigarette	Ultralite	None	None	1, 6
10	USA	America’s Smoke Juice	Korea Electronic Cigarette	Savory	None	None	1, 6
11	USA	None	None	Torque56	None	2015. 07. 08	7, 8, 9
12	USA	None	None	Tribeca	None	2015. 06. 10	7, 8, 9
13	USA	DIY Flavor Shack	DIY Flavor Shack Korea Branch	Paradise	2013. 10. 25	None	10, 11
14	USA	DIY Flavor Shack	DIY Flavor Shack Korea Branch	Cafe Latte	2014. 02. 12	None	10, 11
15	USA	DIY Flavor Shack	DIY Flavor Shack Korea Branch	Pomegranate	2014. 04. 01	None	10, 11
1	Korea	Hanbit Flavor & Fragrance	Maximum Liquid	Washington Duke	2013. 12. 02	24 months	5, 12, 13, 14, 15
2	Korea	Hanbit Flavor & Fragrance	Maximum Liquid	American Blend	2014. 01. 28	24 months	5, 12, 13, 14, 15
3	Korea	Halsol S & F	D&S	Mild Cigar	2014. 03. 20	24 months	None
4	Korea	Halsol S & F	D&S	Texas Cigar	2014. 03. 20	24 months	None
5	Korea	Korea Biomedical	Korea Biomedical	Mild	2014. 04. 24	None	None
6	Korea	Hanbit Flavor & Fragrance	Hello Aromatics	Cigar	2014. 04. 28	12 months	None
7	Korea	None	None	Herb Brown	None	24 months	16, 17, 18
8	Korea	Korea Biomedical	Korea Biomedical	Cig	2014. 05. 09	None	None
9	Korea	Hanbit Flavor&Fragrance	Martha	Cigar	2014. 04. 18	12 months	None
10	Korea	Hanbit Flavor&Fragrance	Martha	Iceblue	2014. 06. 03	12 months	None
11	Korea	Hanbit Flavor & Fragrance	Martha	Applemint	2014. 06. 02	12 months	None
12	Korea	Hanbit Flavor & Fragrance	Martha	Heaven	2014. 06. 03	12 months	None
13	Korea	Hanbit Flavor & Fragrance	Maximum Liquid	Himalaya Frost	2014. 05. 01	24 months	5, 12, 13, 14, 15
14	Korea	Hanbit Flavor & Fragrance	Maximum Liquid	Citronade	2014. 03. 11	24 months	5, 12, 13, 14, 15
15	Korea	Halsol S & F	D & S	Ice Tundra Berry	2014. 03. 20	24 months	None
16	Korea	Halsol S & F	D & S	Blueberry Mojito	2014. 03. 20	24 months	None
17	Korea	Halsol S & F	D & S	Sweet Melon	2014. 03. 20	24 months	None
18	Korea			Pure Nicotine			

1. Smoking damages your health. Once you start smoking, it is very difficult to quit; 2. Tobacco smoke contains the carcinogenic substances Naphthylamine, Nickel, Benzene, Vinyl Chloride, Arsenic, and Cadmium; 3. Not suitable for pregnant or breastfeeding women; 4. Not to be sold to minors; 5. Keep away from children; 6. Smoking causes lung cancer and other diseases. Smoking is harmful to others; 7. This product contains nicotine; 8. Keep locked up and out of the reach of children and pets; 9. Do not drink; 10. It is illegal to sell cigarettes to people under 19; 11. This product is only for use as electronic cigarette nicotine liquid. Do not drink. Avoid contact with the skin; 12. Do not use for any purpose other than its intended use; 13. Never take and do not use the eye; 14. If you experience symptoms that you suspect to be side effects from the use of this product, such as vomiting, headache, *etc.*, please stop using it immediately; 15. Do not use this product if you have an allergic reaction propylene glycol; 16. Do not drink, even if it tastes good; 17. Keep out of reach of children and pets; 18. If the product comes in direct contact with sensitive skin, such as the eyes, ears, or mouth, wash the area thoroughly and consult a specialist.

**Table 2:** Comparison of labeled and measured nicotine concentration values.

No.	Country of Origin	Flavor	Nicotine Concentration (mg/mL)		Difference (%)	*p*-value **
			Labeled	Measured (Mean ± SD, n = 3)		
1	China	Ultralite	2.1	2.1 ± 0.4	0.0	<0.01
2	China	Savory	3.0	3.1 ± 0.6	3.3	
3	China	Torque56	18	12.2 ± 2.7	−32.2	
4	China	Tribeca	18	16.3 ± 0.7	−9.4	
5	Italy	Paradise	18	14.1 ± 2.9	−21.7	
6	Italy	Washington Duke	18	14.8 ± 3.9	−17.8	
7	Italy	American Blend	18	16.9 ± 0.6	−6.1	
8	Netherlands	Mild Cigar	11	6.4 ± 0.7	−30.6	
9	USA	Texas Cigar	NA *	Not Detected		
10	USA	Mild	NA	Not Detected		
11	USA	Cigar	18	17.5 ± 1.3	−2.8	
12	USA	Herb Brown	18	17.3 ± 1.1	−3.9	
13	USA	Cig	16	15.5 ± 0.7	−3.1	
14	USA	Cigar	16	12.1 ± 0.3	−24.4	
15	USA	Citrus Mix	16	12.1 ± 2.0	−24.4	
1	Korea	American Blend	NA	Not Detected		
2	Korea	Cuban Cigar	NA	Not Detected		
3	Korea	Cafe Latte	NA	Not Detected		
4	Korea	Pomegranate	NA	Not Detected		
5	Korea	Cigar	NA	Not Detected		
6	Korea	Iris Melon	NA	Not Detected		
7	Korea	The Black	NA	Not Detected		
8	Korea	Korean Mini	NA	Not Detected		
9	Korea	USA Mix	NA	Not Detected		
10	Korea	Iceblue	NA	Not Detected		
11	Korea	Applemint	NA	Not Detected		
12	Korea	Heaven	NA	Not Detected		
13	Korea	Himalaya Frost	NA	Not Detected		
14	Korea	Citronade	NA	Not Detected		
15	Korea	Ice Tundra Berry	NA	Not Detected		
16	Korea	Blueberry Mojito	NA	Not Detected		
17	Korea	Sweet Melon	NA	Not Detected		
18	Korea	Pure Nicotine	NA	150.3 ± 7.9	Not applicable	

* NA: not available; **p-value obtained from Wilcoxon signed rank sum test.

## 4. Discussion

In this study, we found that the labeling of e-liquids, including manufacturing date, expiration period, and health warning statement, from both imported and domestic manufacturers sampled from South Korean retailers was not standardized. Specifically, we noticed that none of the imported liquid refill products had manufacturing dates. We also found that 70% of domestic products did not have a health warning statement, while 80% of imported products did. The types of health warning statements varied depending on the manufacturer or supplier. In this study, the measured nicotine concentration was significantly lower than the labeled nicotine concentrations in the refill products (*p* < 0.01) which was supported by a recent study [8]. However, a large number of other studies have found more nicotine, compared to labeled concentrations [9,10]. 

Our findings of inaccurate nicotine concentration of refill products should be taken into careful consideration. Consumers can face the risk that they do not know their absorption-dose of nicotine through smoking e-cigs. During our purchasing process, we found that liquid refill products could be mixed with liquid nicotine from a separate bottle by a clerk at the consumer’s request, which raises the risk of harm due to an uncontrolled or inaccurate dose of nicotine. These findings raise safety concerns for not only Korean current and potential users, but other public health professionals [11]. 

Also, the concentration (150 mg/mL) of one product labeled “pure nicotine”, raises concerns since it may be poisonous to consumers, especially children, who may accidentally ingest it. A study has reported that an estimated level of 10 mg nicotine can be fatal to children [12]. In April 2014, the U.S. Food and Drug Administration released a framework for federal regulations, calling for warning labels on packaging, as well as a ban on selling e-cigs to children [13]. 

Through an international collaboration we objectively found that the nicotine content of e-cig refill bottles was close to the concentration stated on the labels which were much improved results, compared to previously conducted studies [11,14]. However, we found that the level of quality control in nicotine contents was not enough; some products made in Italy or the U.S. contained less nicotine than the labeled amount. Although our methodology allows the quantitative analysis of nicotine concentrations in e-cig refill products, a recent study reported that the pH values for e-cig refill products correlated with the measured total nicotine concentration [15]. As we mentioned earlier, most of our refill products contained tobacco flavor additives that might influence the resulting e-liquid pH, possibly creating a weaker nicotine/pH relationship [15]. Thus, measured nicotine levels were likely underestimated. Future studies should expand the examination of the discrepancies between labeled and actual nicotine concentration values to a wider range of products in a spectrum of pH, flavors and from various manufacturers, suppliers, and retail shops, because there may be considerable variability within and between them. In this study, there were several limitations. Due to funding and time constraints, we could not investigate all products on the market. In addition, a couple of products were purchased without a box. Therefore, label information was summarized based on the information stated directly on the bottles. More information, such as health warning statements, might have been available on the box, but not on the bottle. However, according to our experiences, the fact that many products can potentially be purchased without a box (and thus, without label information) is noteworthy, since this is the way most consumers receive the product. Future studies with larger samples will be useful to evaluate discrepancies between nicotine content and label information within and between companies.

There is no denying the surging popularity of e-cigs among Korean adolescents [16] as well as among young people in other countries [17,18]. As a precautionary principle, labeling accuracy for e-cigs, including nicotine content, date of manufacture and health warning statements, is of growing importance. The effectiveness of e-cigs as a less harmful nicotine delivery system is still in question with competing views on the use of such products as gateways to tobacco use or as a type of nicotine replacement therapy [19] and the benefits and risks of their use are still being vigorously debated globally [20]. Nevertheless, standards for the labeling of e-cig liquid refill products, including accurate nicotine content, roper health warning statements, date of manufacture and expiration date, should be applied to all such products, including pure nicotine sold in South Korea. This report should serve as a basis for the development of product regulations in South Korea. 

## 5. Conclusions 

This study revealed the urgent need for national labeling standards for these products and necessity of measurement of concentration levels of other toxic or carcinogenic compounds in the liquids, as well as in the vapors.
